# Feasibility and effects of applying stochastic resonance whole-body vibration on untrained elderly: a randomized crossover pilot study

**DOI:** 10.1186/s12877-015-0021-4

**Published:** 2015-03-12

**Authors:** Slavko Rogan, Lorenz Radlinger, Roger Hilfiker, Dietmar Schmidtbleicher, Rob A de Bie, Eling D de Bruin

**Affiliations:** Bern University of Applied Sciences, Health, Bern, Switzerland; HES-SO // University of Applied Sciences Western Switzerland; Valais, Sion, Switzerland; Department of Sport Science, Wolfgang-Goethe University Frankfurt, Frankfurt, Germany; Department of Epidemiology, Maastricht University, CAPHRI School for Public Health and Primary Care, Maastricht, The Netherlands; Centre for Evidence Based Physiotherapy, Maastricht University, PO Box 616, 6200 MD Maastricht, The Netherlands; Department of Health Sciences and Technology, Institute of Human Movement Sciences and Sport, ETH Zurich, Wolfgang-Pauli-Str. 27, HIT J 31.2, CH-8093 Zurich, Switzerland

**Keywords:** Feasibility, Adherence, Attrition, Balance, Reaction time

## Abstract

**Background:**

Aging is associated with loss of balance and activity in daily life. It impacts postural control and increases the risk of falls. The current study was conducted to determine the feasibility and long-term impact of stochastic resonance whole-body vibration (SR-WBV) on static and dynamic balance and reaction time among elderly individuals.

**Methods:**

A randomized crossover pilot study with blinding of the participants. Twenty elderly were divided into group A (SR-WBV 5 Hz, Noise 4/SR-WBV 1 Hz, Noise 1) or group B (SR-WBV 1 Hz, Noise 1/SR-WBV 5 Hz, Noise 1). Feasibility outcomes included recruitment, compliance and safety. Secondary outcomes were Semi-Tandem Stand (STS), Functional Reach Test (FRT), Expanded Timed Get Up-and-Go (ETGUG), walking under single (ST) & dual task (DT) conditions, hand and foot reaction time (RTH/RTF). Puri and Sen Rank-Order L Statistics were used to analyse carry-over effects. To analyse SR-WBV effects Wilcoxon signed-ranked tests were used.

**Results:**

With good recruitment rate (55%) and compliance (attrition 15%; adherence 85%) rates the intervention was deemed feasible. Three participants dropped out, two due to knee pain and one for personal reasons. ETGUG 0 to 2 m (p = 0.143; ES: 0.36) and ETGUG total time (p = 0.097; ES: 0.40) showed medium effect sizes.

**Conclusions:**

Stochastic resonance training is feasible in untrained elderly resulting in good recruitment and compliance. Low volume SR-WBV exercises over 12 training sessions with 5 Hz, Noise 4 seems a sufficient stimulus to improve ETGUG total time. The stimulation did not elicit changes in other outcomes.

**Trial registration:**

This trial has been registered at the U.S. National Institutes of Health under ClinicalTrials.gov: NCT01045746.

## Background

Postural balance skills of the elderly become increasingly limited due to normal or pathological ageing [1]. These age related limitations in balance skills may be explained by changes in muscle mass, decreased reflex activity, mobility impairments, loss of somatosensory sensors as well as being due to an impairment of central processing, a deficit of motor response functions and a reduction in the functioning of the vestibular and visual systems [2,3]. These limitations are, furthermore, most likely associated with risk of injury or risk of falls [4-6]. A sedentary lifestyle in elderly individuals further increases the risk of falling whereas physically active elderly have a reduced risk, especially for falls resulting in injuries [7].

Despite the fact that physical activity (PA) for elderly is one of the major elements for general health prevention, too few elderly engage in PA [8]. Inactive or sedentary elderly should, therefore, be motivated to increase their PA [9]. It is important, however, to consider low baseline fitness and mobility levels in pre-frail or frail or rather untrained elderly when starting an exercise program. Elderly individuals with low baseline fitness and mobility levels who want to start a training program should start with an exercise program that meets their physical capabilities [10]. These individuals are advised to first enter a “skilling up” phase before more traditional forms of training are implemented [5] in case their capabilities are low. The question of what kinds of exercises are appropriate for “skilling up”, however, remain to be explored [11]. With this insight, trainers may prescribe balance exercises more effectively for untrained and frail elderly with different physical activity backgrounds who have impairments in static, dynamic or functional balance skills.

Systematic reviews concluded that, compared to more demanding interventions, whole body vibration (WBV) might be a more safe and less-fatiguing type of exercise [12] with beneficial effects on dynamic balance skills. Pilot studies showed that an intervention with stochastic resonance WBV (SR-WBV) in the elderly is both safe and feasible [13,14] and has positive effects on physical functioning [15]. The use of SR-WBV might, therefore, be valuable for untrained or frail elderly where the neuromuscular systems might not be able withstanding higher loading and long training sessions [16].

There are two types of WBV devices on the market [17]: sinusoidal (i.e. with a constant vibration frequency) and stochastic resonance vibration (i.e. with random vibration frequencies and harmonics) [18]. During sinusoidal WBV participants stand on a single plate platform that vertically or side alternating vibrates with high frequency. Frequencies range between 20 to 50 Hz and amplitudes between 2 to 14 mm. Stochastic resonance whole-body vibration (SR-WBV) devices vibrate with frequencies between 1 and 12 Hz and amplitudes between 3 and 6 mm while the feet of the participants are placed on two independently powered stochastically resonating vibrating platforms [17].

However, there is a lack of evidence concerning the feasibility of implementing such exercise interventions in a primary training program aimed at “skilling up” of untrained elderly. New treatments usually have to go through a series of phases to test whether they are safe and effective [19] before larger scale studies and application in clinical practice are to be considered. The aim of this pilot study was to perform a phase II trial according the model for complex interventions advocated by the British Medical Research Council [20] to test the feasibility and effects of a SR-WBV program in a group of untrained elderly. The study aimed to (1) develop an exercise intervention based on principles of exercise theory and to deliver it to untrained elderly, (2) evaluate the feasibility of the intervention and the ability to recruit and retain elderly individuals, and (3) assess whether the treatment had some effect on physical performance.

## Methods

### Design

**Figure 1:**
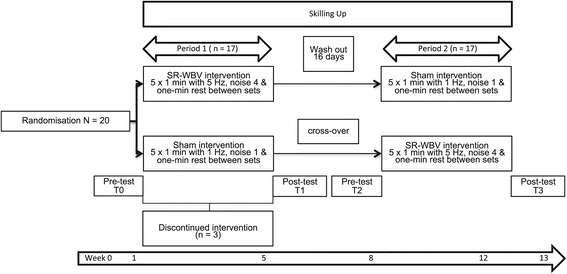
**Flow chart of this cross-over pilot study.**

The study used a crossover research design, involving 2 randomised groups (groups A and B) with blinding of the participants (Figure 1). The SR-WBV exercise was supervised and performed in a home-for-the-aged (Senevita Residenz Multengut, Muri, Switzerland), however, focused on individuals comparable with older community-dwellers independent in mobility functions and not on individuals comparable to nursing home residents. Group A started with the intervention while group B received a sham intervention. Treatments were reversed for the groups following a wash-out period. The assessor was not blinded to group allocation. The participants were familiarized with the treatment protocol one week prior to data collection.

### Participants

Participants were included when fulfilling the following criteria: age over 65 years, able to stand with or without walking aids, classified as being only lightly dependent on nursing care according the BESA classification level 0, 1, 2^a^, living in the Canton of Berne and a having a score >22 in the Mini-Mental Status Examination (MMSE) Test. Exclusion criteria were: visual disturbances^b^, lower or upper leg prosthesis, acute joint disease, acute thrombosis, acute fractures, acute infections, acute tissue damage, acute surgical scars or alcohol abuse.

### Randomisation

Randomisation was performed by an independent research assistant. The participants were stratified by sex and were randomly assigned to either group A or group B. The blinded independent research assistant guaranteed concealed allocation sequence through the use of numbered sealed opaque envelopes distributed after the completion of all baseline assessments.

Written consent was obtained from all participants before enrolment in the study. The study protocol was approved by the Ethical Committee of Canton Berne (No.228/09) and was based on the declaration of Helsinki and registered under the U.S. National Institutes of Health (https://clinicaltrials.gov/) trial Number NCT01045746.

### Protocol

**Figure 2:**
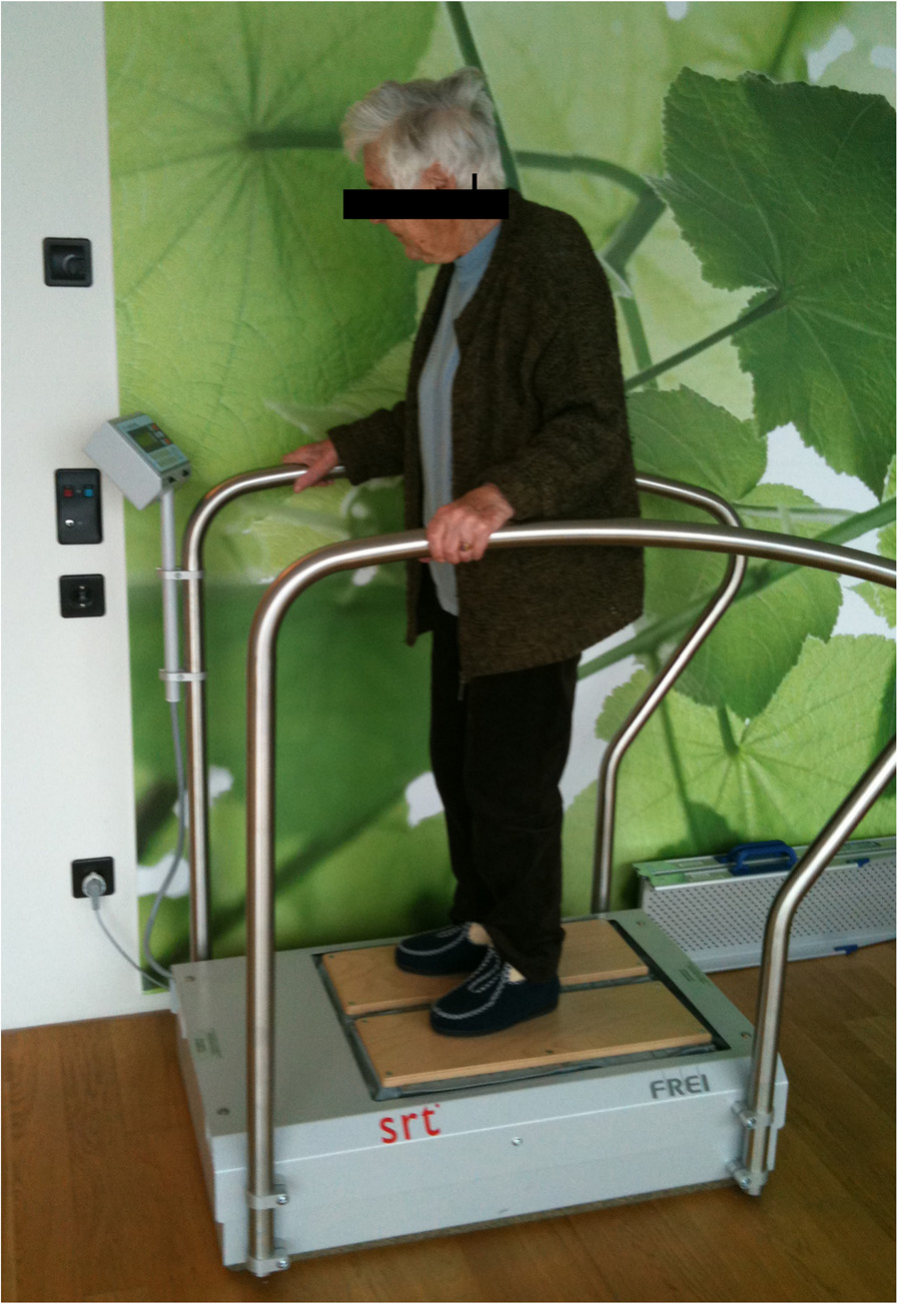
**Picture of the Zeptor med® device.**

The participants were exposed to SR-WBV using a Zeptor med® device (Frei Swiss AG, Zurich, Switzerland) containing two three dimensionally vibrating plates (Figure 2). The participants were familiarized with the vibration treatment one week prior to the experiment. They stood freely on both legs wearing comfortable shoes. The participants were instructed to maintain a standing position with slight flexion of the hips, knees and ankle joints. In period 1, the participants in group A received 5 sets of 1 minute SR-WBV with 5 Hz, Noise 4 with 1 minute of rest between sets, three times a week, during four weeks. A minimum of one day rest in between training sessions was warranted. Participants in group B received a sham intervention of 5 sets of 1 minute SR-WBV with 1 Hz, Noise 1, where the 1 Hz frequency condition can be expected to have no training effect [14]. After a wash-out period of 16 days, period 2 started. Group A received the sham intervention of 5 sets of 1 minute SR-WBV with 1 Hz, Noise 1 and group B received 5 sets of 1 minute SR-WBV with 5 Hz and Noise 4 during four weeks. The primary and secondary outcome variables were measured at baseline (T0) before training, after four weeks of training (T1) in period 1, and after the second four weeks intervention period (T3).

### Outcomes

#### Recruitment rate, attrition, and program adherence

The criteria for success [19] of this pilot study were based on feasibility and focused on recruitment, attrition and adherence to the stochastic resonance WBV intervention. Recruitment of a third of the residents deemed eligible for the training, a 15% attrition rate, and 80% attendance rate [21] for the training were deemed acceptable.

For recruitment, data for the total sampling frame (both those approached and not approached) for inclusion in the trial were taken to assess generalizability to all elderly individuals within the facility. We measured the inclusion rate—i.e. the proportion of participants invited to participate who enrolled into the study—and distinguished between those who refused, did not respond or who were willing but excluded (volunteered but did not meet the study inclusion criteria). For attrition, we measured the number of participants lost at follow-up. For adherence to the intervention we recorded engagement with the intervention, e.g. compliance with all trainings. There were a total of 24 stochastic resonance WBV training sessions possible for each individual.

### Secondary outcomes

Semi-Tandem Stand (STS) was measured using a multi-component force platform (Kistler, Typ 9286BA, Winterthur, Switzerland). STS measurement with this approach has shown to be reliable [22]. Each platform signal was transformed in an amplifier, digitally sampled with 1 kHz using a 12-bitA-/D-converter (Meilhaus, ME-2600i, SisNova, Engeneering, Zug, Switzerland) and analysed using ADS-Software-Program 1.12 (uk-labs, Kempen, Germany). The participants were instructed to perform STS for 20 seconds on the force platform. They were positioned by placing their right foot in the right upper quadrant and the left foot in the left lower quadrant of a custom built cadre that was removed after positioning. The arms were in a neutral position at the side of the body. The participants had to look straight ahead and fix their focal viewpoint on a green marker positioned at eye-level at a distance of 3 meters. Anterior-posterior (AP) and medial-lateral (ML) sway during the STS was calculated from force-time curves. The test was repeated twice with a rest of 1 minute in between.

Functional Reach Test (FRT) was used to quantify dynamic balance [23]. This test is both valid and reliable [24] for the target population. A yardstick is attached to a wall at about shoulder-height. The participant stands facing the wall, with the arm in 90° anteflexion, and reaches maximally forward without moving the feet. The FRT has been associated with an increased risk of falls and frailty in elderly unable to reach more than 15 cm forward [23]. The measurement data were obtained from the best three attempts out of five and averaged.

The Expanded Timed Get Up-and-Go (ETGUG), a reliable clinical assessment, measured time series of functionally important tasks using a multimemory stopwatch [25,26]. The participants were asked to rise from a chair, to walk at their normal speed to the end of the walkway, to turn around and to walk and sit back on the chair. At 2, 8 and 10 meters along the walkway, markers were set (using coloured tape on the floor), allowing measurement of the mean times. Measurements were conducted using a digital hand stopwatch (Timex: Ironmen Triathlon, Middlebury, CL, USA). The test was repeated twice with minimum of one minute rest in between.

Gait of each participant was assessed during usual walking at preferred velocity under single and dual task conditions over a distance of 20 m in a corridor of the Senevita Residenz Multengut Muri (Switzerland) with a digital hand stopwatch (Timex: Ironmen Triathlon, Middlebury, CL, USA). The test was repeated twice with a minimum of one minute rest in between. Gait assessment at preferred velocity informs about actual, subject-specific behaviour [27] which is indicative of a decrease in the performance of gait in senior adults who have fallen and may be related to diminished strength, balance, and tactile sensation [28]. The measurement device and testing protocol have previously been described and identified as being reliable in older adults [29].

Simple reaction time was measured from both hand (RTH) and foot (RTF) to measure psychomotor speed in milliseconds using a hand-held electronic timer and a light as the stimulus and depression of a switch by the finger and the foot as the responses [30,31]. Participants performed 5 practice and 10 experimental trials.

For safety reasons, the participants were interviewed before and immediately after vibration training on their well-being, feelings of (in)stability and for adverse effects such as dizziness and pain during vibration. Discontinuations of participation in the study were noted.

### Statistical analysis

This pilot study used nonparametric statistical analyses. Mann–Whitney *U* test assess baseline characteristics (T0) and treatment effects between group A and group B. Puri and Sen L Statistics for Ranked Data [32] analysed carry-over effects. Carry-over effects were compared with a two-factorial analysis of variance with repeated measures [33]. Pillai’s Trace was used to calculate L. In case a carry-over effect was present, the data were analysed with the first period data only: e.g., similar to a parallel design [33]. In case no carry-over effect was present, treatment effects were calculated with Mann–Whitney *U* test from both periods. The difference in mean from period 1 minus period 2 was compared between the groups [34].

The results are reported as an estimate of the intervention effect as mean ± SDs. P-values < 0.05 were considered significant. All analyses were conducted using SPSS Version 19.0 for Windows (SPSS Inc., Chicago, IL, USA) and the statistical function of the Microsoft® Excel® 2008 for Mac Version12.2.7 software. The magnitude of effects were calculated and expressed as r = Z/√N. For r an effect size of 0.1 is considered a “small” effect, around 0.3 a “medium” effect and 0.5 and above, a ‘large’ effect [35]. In addition, participants’ compliance to the treatment protocol was calculated using the following formula: Number of vibration sessions ÷ the total number of possible vibration sessions x 100.

The program G*Power 3 was used for the post hoc calculation of power (www.psycho.uni-duesseldorf.de/abteilungen/aap/gpower3/). The CONSORT 2010 guidelines regarding randomised trials (www.consort-statement.org) and recommendations of items to include when reporting a pilot study [19] were followed for describing the results of this pilot.

## Results

Figure 1 describes the flow of the participants through the study. Socio-demographic and anthropometric characteristics are summarized in Table 1. None of the participants reported any injuries or medical conditions that could affect their balance. Participants reported to being generally healthy.

**Table 1:** **Demographic characteristics and baseline values (mean ± SD)**

	**Group A**	**Group B**	**p**
	**(n = 10)**	**(n = 10)**	
Age (years)	76.8 ± 7.7	80.7 ± 5.7	0.290
Height (m)	1.76 ± 0.07	1.64 ± 0.05	0.001
Weight (kg)	81.00 ± 10.4	69.20 ± 9.8	0.034
BMI (Kg/cm^2^)	26.1 ± 2.5	25.8 ± 3.8	0.597
Sway AP (mm)	36.5 ± 6.7	30.1 ± 12.0	0.162
Sway ML (MM)	32.9 ± 9.1	26.8 ± 15.0	0.199
FRT (cm)	33.2 ± 7.2	28.3 ± 8.1	0.174
ETGUG ss (s)	2.20 ± 1.0	2.72 ± 1.3	0.226
ETGUG 0–2 m (s)	1.67 ± 0.7	2.05 ± 1.2	0.472
ETGUG 2–8 m (s)	3.46 ± 0.9	3.69 ± 2.2	0.364
ETGUG turn (s)	3.15 ± 0.9	3.87 ± 1.4	0.151
ETGUG 12–18 m (s)	4.10 ± 0.9	5.18 ± 2.1	0.121
ETGUG 18–20 m (s)	2.20 ± 0.8	2.19 ± 0.8	0.112
ETGUG total time (s)	16.8 ± 3.4	20.1 ± 7.0	0.096
ST (m/s)	0.77 ± 0.2	0.88 ± 0.3	0.705
DT (m/s)	0.85 ± 0.3	1.1 ± 0.4	0.082
RTH (ms)	276 ± 0.7	281 ± 0.7	0.791
RTF (MS)	299 ± 0.6	331 ± 0.8	0.545

### Recruitment, attrition, and adherence

The facility had a total of 100 residents from which staff representatives estimated 65 fulfilled eligibility criteria and, therefore, represented the potential sampling frame. Two information sessions were held and attended by 45 residents. From these 45 persons, 25 persons were deemed eligible and were invited to participate. 20 eligible persons (10 women, 79.85 ± 6.6 years and 10 men, 78 ± 7.3 years) were recruited and enrolled in the study resulting in a recruitment rate of approximately 55%. Inclusion rate—i.e. the proportion of participants invited to participate who enrolled—was 80%. The participants were willing to be randomized. Seventeen elderly individuals participated at follow-up measurements that resulted in a 15% attrition rate. Three participants dropped out during the training sessions. Two participants discontinued training due to knee pain unrelated to the training and one for personal reasons (Table 2). The number of SR-WBV sessions completed divided by the possible training sessions was 95%, leading to excellent adherence to the study protocol over the four weeks training periods. Neither subjective nor objective side-effects related to the used intervention were reported.

**Table 2:** **Overview for repeated measures Puri & Sen-analyses of ranked data for cross-over effect**

	**Pillai’s trace (r** ^**2**^ **= SS**_**Bet**_ **/SS**_**Tot**_ **)**	**L [(N-1) r** ^**2**^ **]**	**Probability**
Sway AP (mm)	0.038	0.278	0.761
Sway ML (mm)	0.044	0.321	0.731
FRT (cm)	0.255	2.394	0.128
ETGUG (s) ss	0.291	2.877	0.090
ETGUG (s) 0–2 m	0.062	0.467	0.637
ETGUG (s) 2–8 m	0.175	1.485	0.260
ETGUG (s) turn	0.009	0.066	0.936
ETGUG (s) 12–18 m	0.239	2.201	0.148
ETGUG (s) 18–20 m	0.064	0.482	0.628
ETGUG (s) total time	0.273	2.626	0.108
ST (m/s)	0.156	1.295	0.305
DT (m/s)	0.158	1.134	0.300
RTH (ms)	0.022	0.159	0.855
RTF (ms)	0.271	2.607	0.109

Legends: AP: anterior-posterior, ML: medial-lateral, FRT: Functional Reach Test, ETGUG: Expanded Timed Get Up-and-Go, ST: single task, DT: dual task, RTH: reaction time hand, RTF: reaction time foot, mm: millimetre, s: seconds, m/s: metre/seconds, ms: milliseconds, ss: sit-to-stand, m: metre.

### Secondary outcomes

No carry-over effect was found for any of the outcomes measures (Table 2). Table 3 summarizes the main outcome results for all outcome measures. ETGUG 0 to 2 m (p = 0.143; ES: 0.36) and ETGUG total time (p = 0.097; ES: 0.40) showed no significant changes albeit medium effect sizes. The other values presented no significant changes combined with small effect sizes.

**Table 3:** **Difference values from group A and B in mean ± SD**

	**Group A**	**Group B**	**P**	**ES**
	**Difference period 1 - 2**	**Difference period 2 - 1**		
**Sway ML (mm)**	- 11.73 ± 4.6	- 10.23 ± 6.3	0.435	0.00
**Sway AP (mm)**	- 6.91 ± 4.5	- 11.79 ± 5.6	1.000	0.19
**FRT (cm)**	- 12.67 ± 14.6	- 2.33 ± 0.8	0.432	0.19
**ETGUG (s) ss**	1.29 ± 0.5	1.39 ± 0.3	0.770	0.06
**ETGUG (s) 0-2 m**	- 0.39 ± 0.7	- 0.07 ± 0.2	0.143	0.36
**ETGUG (s) 2-8 m**	- 0.54 ± 0.2	- 0.99 ± 0.4	0.435	0.19
**ETGUG (s) turn**	- 0.14 ± 0.1	0.04 ± 0.2	0.626	0.12
**ETGUG (s) 12-18 m**	- 0.20 ± 0.2	0.02 ± 0.2	0.495	0.17
**ETGUG (s) 18-20 m**	- 0.20 ± 0.1	- 0.23 ± 0.2	0.696	0.09
**ETGUG total time (s)**	- 0.13 ± 0.3	1.15 ± 0.4	0.097	0.40
**ST (m/s)**	- 0.02 ± 0.02	- 0.01 ± 0.2	1.000	0.00
**DT (m/s)**	- 0.02 ± 0.01	0.02 ± 0.02	0.329	0.24
**RTH (ms)**	- 0.008 ± 0.009;	- 0.005 ± 0.01	1.000	0.00
**RTF (ms)**	- 0.010 ± 0.005	- 0.010 ± 0.01	0.329	0.24

Legends: Difference values from period 1 - period 2. *P*-values were computed using Wilcoxon signed rank test for group 1 and group 2 at period 1 and period 2.

ES: effect size, AP: anterior-posterior, ML: medial-lateral, FRT: Functional Reach Test, ETGUG: Expanded Timed Get Up-and-Go, ST: single task, DT: dual task, RTH: reaction time hand, RTF: reaction time foot, mm: millimetre, s: seconds, m/s: metre/seconds, ms: milliseconds, ss: sit-to-stand, m: metre.

## Discussion

This randomized cross-over pilot study tested the feasibility of SR-WBV training applied to untrained elderly living in a home-for-the-aged. Furthermore, this study investigated the effects of a four-week SR-WBV training on static and dynamic balance and reaction time. The main findings showed that a randomised controlled cross-over trial with SR-WBV is both feasible and safe for untrained elderly. Those individuals that responded and visited an information session showed a large inclusion rate and in majority remained in the intervention until completion. These findings indicate the importance of information sessions for elderly individuals where questions and concerns about new interventions can be met.

This pilot study provided useful information about the feasibility of the experimental intervention that used SR-WBV for “skilling-up” training. Our participants tolerated the SR-WBV intervention. They were also able to progress in intensity and duration of the exercises. However, our experience suggests that our SR-WBV component was not of sufficient duration and/or intensity to ameliorate physical functioning capacity as indicated by no improvements in the secondary outcomes. Neither group showed improvements or tendencies towards improvement in any of the outcome parameters within the program duration of four weeks. This finding might be attributed to a lack of power due to the sample size used or is due to the time frame of the intervention. Kawanabe and colleagues [36] indicated that effects of WBV training in the elderly may be expected after a two months study period. We believe, therefore, that it is feasible to proceed to a sufficiently powered main study only with major modifications to the protocol; e.g. adapt the intensities and/or length of training. It might well be, for example, that we should increase the frequency of the SR-WBV vibration. The current pilot study used a frequency with 5 Hz, Noise 4. Haas [37] and Turbanski [38] used in their studies effective frequencies of 6 Hz. In a future study, the amplitude of vibration should possibly be 6 Hz.

Summarising the findings and limitations of this study it becomes clear that this study only reveals first estimates for the chosen outcome measures. We implemented a strict study design to control threats to validity. A next step would be to replicate the findings in a new exercise group of institutionalised elderly individuals as an additional control procedure. Although we are aware of the fact that the emphasis of a pilot study should be placed on feasibility and not on statistical significance [19] our data allow for a sample size calculation for a future trial. To avoid a type I or II error in this future trial we need, based on our observed value for the ETGUG total time (with values of the last training of EXPERIMENTAL = 13.5 ± 3.1 s; CONTROL = 19.3 ± 7.9 s), an estimated sample size of 50 participants per group for a two group pretest-posttest design. This would result in 80% power at an α-level of 0.05 and is based on the assumption that the standard deviation of the response variable is 5.5. To account for attrition over time, the required sample size should increase by 15%. It should be stressed, however, that this sample size calculation should be interpreted with caution because our estimates may be unrealistic or biased because of the limited sample size [19].

## Conclusions

We conclude that pilot studies with explicit feasibility objectives and success criteria are important foundation steps in preparing for large trials [19] and for development of Rehabilitation research programs [39]. Ongoing formal review of the multifaceted issues inherent in the design and conduct of pilot studies can provide invaluable feasibility and scientific data for rehabilitation specialists, e.g. physiotherapists, willing to perform clinical trials [40] and may also be highly relevant for furthering the development of theory based rehabilitation [39]. SR-WBV training is feasible and, although not showing significant effects, shows trends to stronger improvement in the overall time to complete a series of functionally important tasks as assessed with the ETGUG with a medium to large effect size of 0.4. The application in a main study is deemed feasible, however, with a need for protocol modifications. A minimum of ± 55 participants per group are required to achieve a power of 80% at the 5% level of significance based on ETGUG total time and considering the expectable attrition rate in a required larger scale study. This study encourages the further development of this intervention, preferably with a randomized control design.

## Endnotes

^a^BESA and its assessment instruments are based on the results of scientific research. Thanks to the four steps of BESA – clarification of the available resources, agreements concerning the aims of health care, taxation of the costs and improvement of the quality level of health care – the main elements of the process of health are systematically sustained. BESA is actually used in more than 400 homes or residences for elderly people in Switzerland.

^b^Visual disturbances are abnormalities of sight and associated with neurological disorders (diabetes), often include double vision, moving vision like nystagmus, blindness or reduced view field.

## Abbrevations

AP: Anterior-posterior sway
DT: Dual task
ES: Effect size
ETGUG: Expanded timed get up-and-go
FRT: Functional reach test
ML: Medial-lateral sway
MMSE: Mini-mental status examination
PA: Physical activity
RTF: Foot reaction time
RTH: Hand reaction time
STS: Semi-tandem stand
SR-WBV: Stochastic resonance whole-body vibration
WBV: Whole-body vibrationa
